# Strangulated penis in a prison inmate: Complete penile degloving and full‐thickness skin graft

**DOI:** 10.1002/iju5.12663

**Published:** 2023-11-01

**Authors:** Abdulla Uthman, Shahad Al‐Mashhadani, Naomi Blower, Muhammed Iqbal

**Affiliations:** ^1^ Cwm Taf Morgannwg University Health Board Pontylun UK; ^2^ Baghdad Teaching Hospital Baghdad Iraq

**Keywords:** penile necrosis, skin graft, strangulated penis

## Abstract

**Introduction:**

Penile strangulation is a rare urological emergency that necessitates urgent management. The reasoning behind it may include sexual pleasure, erection reinforcement, or a psychiatric disorder.

**Case presentation:**

Despite being an uncommon complication of penile strangulation, we report a 45‐year‐old prison inmate who presented with penile shaft necrosis secondary to using a non‐metallic constriction object. The patient reported a 5‐day history of progressive penile pain, edema, and skin injury but no urinary symptoms. The patient underwent complete penile skin degloving, circumcision, and insertion of a suprapubic catheter. Postoperatively, the penile tissue appeared healthy, and the wound was granulating. On the eleventh day following degloving, a full‐thickness skin graft was taken from the groin area. The patient remained in the hospital for 20 days, during which he was clinically stable with clean, healing wounds.

**Conclusion:**

Early management of penile strangulation is vital in order to prevent vascular and mechanical complications.

**Abbreviations & Acronyms**
EUA: examination under anesthesia
FTSG: full‐thickness skin graft
SPC: suprapubic catheter

**Keynote message**

- Penile strangulation is a rare urological emergency that requires urgent management.
- Early management of penile strangulation is essential to prevent vascular and mechanical complications.
- Patients who experience penile strangulation should undergo a psychological assessment to evaluate potential underlying psychiatric disorders that may contribute to the incident.

## Introduction

Penile strangulation is a rare urological emergency that requires urgent management. Several single case reports have been published in the literature, with the reason behind it being sexual pleasure, erection reinforcement, or secondary to a psychiatric disorder.^1^

Necrosis is an uncommon complication of penile strangulation. This is because each corpus cavernosum has its own arterial supply. Furthermore, Buck's fascia and the corporeal tissue are thick and can bear pressure on the corporeal arteries.^2^

However, we present a case of a 45‐year‐old male prison inmate who presented with penile shaft necrosis secondary to using a non‐metallic penile constriction object.

## Case presentation

A 45‐year‐old male prisoner presented with necrosis on the penile shaft secondary to using a non‐metallic penile constriction object. The patient reported a 5‐day history of progressive penile pain, edema, and skin injury but no urinary symptoms. There was no notable medical background or history reported.

**Fig. 1:**
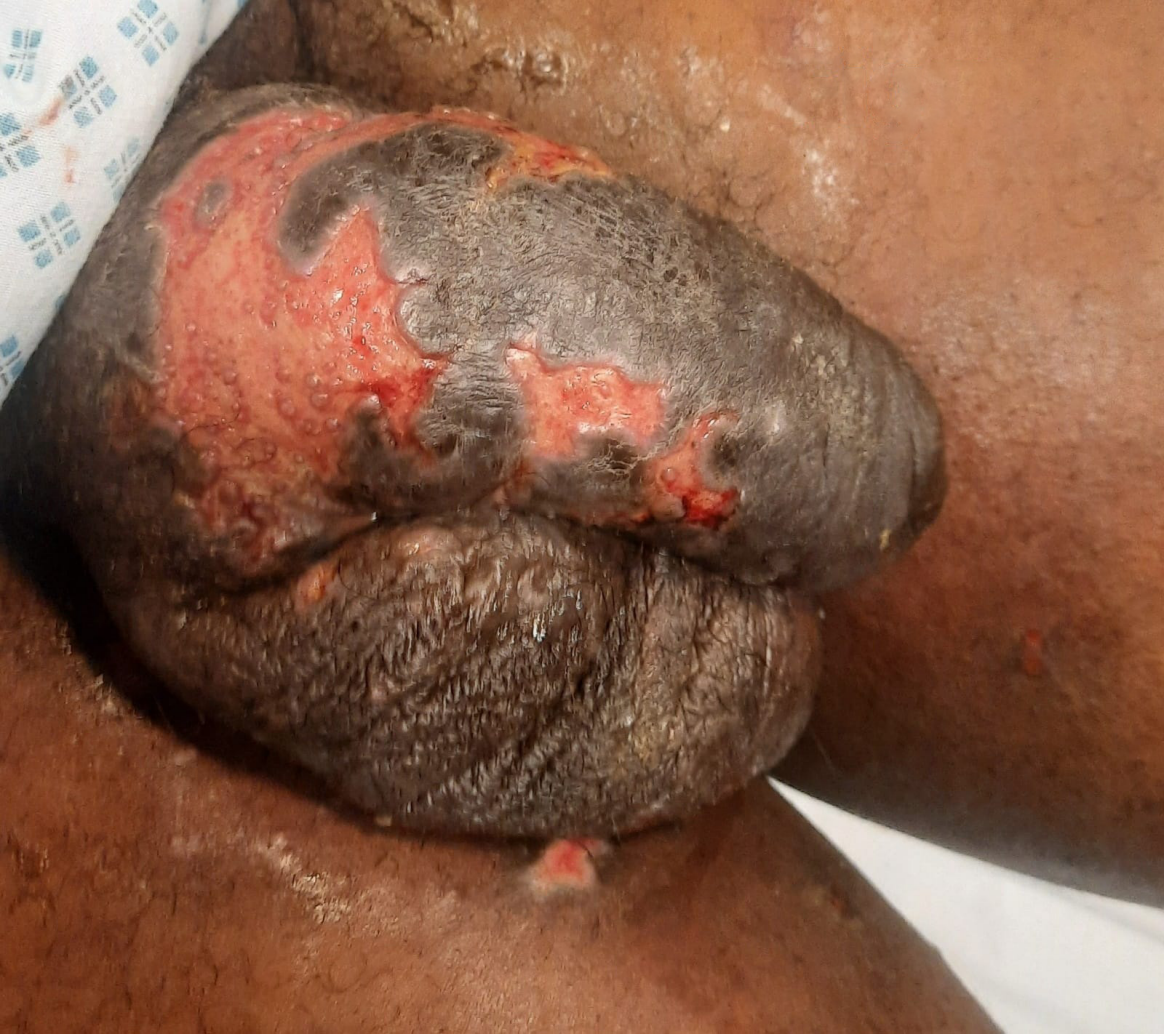
Penile shaft shows signs of necrosis, with infected necrotic skin, pus exudate, and patches of missing dermis on both the dorsal and ventral aspects.

Upon penile examination, the patient showed signs of malodor, purulent exudate, infected necrotic skin, and missing dermis on the dorsal and ventral aspects of the penile shaft (Fig. 1). The distal penis was edematous and tender. The patient's vital signs were stable, and laboratory investigations were normal, with no fever present.

**Fig. 2:**
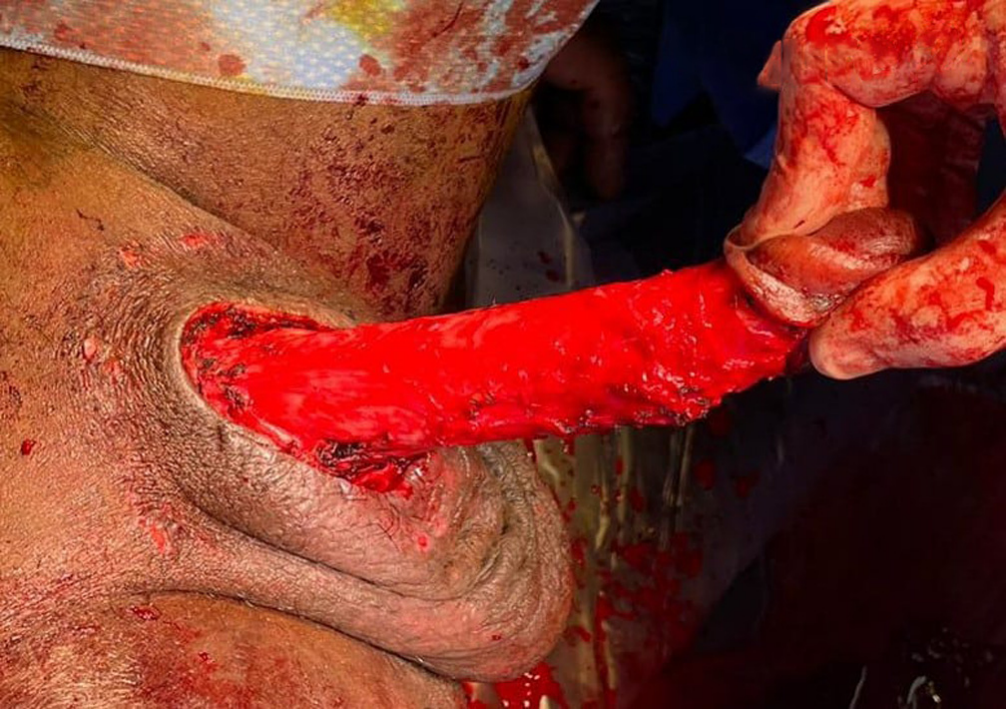
Patient underwent circumcision and skin degloving, resulting in the complete removal of skin from the glans edge to the penile base, as well as midline anterior scrotal skin. The Buck's fascia remained intact, and the SPC was in place.

Immediate treatment involved prescribing a combination of cephalosporin, gentamicin, and metronidazole, along with potent analgesia. Prompt operative management was then undertaken, which included urgent EUA, rigid cystoscopy, SPC insertion, and complete penile skin degloving. The procedure was performed under general anesthesia in the Lloyd Davis position, and a 16 Fr SPC was inserted under cystoscopy guidance. Complete skin degloving from the glans edge to the penile base and midline anterior scrotal skin was undertaken, along with circumcision. Buck's fascia was found to be intact (Fig. 2). Following the procedure, a Jelonet dressing, blue gauze, and crepe bandage were applied.

No early postoperative complications were reported, and the patient's laboratory investigations were normal. The patient remained afebrile and had stable vital signs. The patient was continued on the same antibiotics regimen.

On the third‐ and seventh‐day post‐penile degloving, the patient had EUA, which revealed no necrotic tissue or infection. The penile tissue was healthy, and the wound was granulating. The penile wound was irrigated with peroxide, iodine, and saline and redressed. The microbiology team advised starting the patient on meropenem and clindamycin based on the penile skin microbiology results which showed the presence of Staphylococcus aureus and Beta‐haemolytic streptococcus.

On the eleventh day following penile degloving, a FTSG was performed from the groin area in a joint procedure involving the urology and plastic surgery teams. The wounds on the penile shaft and scrotum were found to be granulating and healthy. The wound edges and base were refreshed, and minimal excision of irregular benign subcutaneous tissue was performed. Hemostasis was achieved, and the wound was washed out with chlorhexidine and saline.

Scrotal skin was mobilized with a sub‐dartos layer to enable scrotal wound closure in layers. A urethral catheter was inserted to protect the urethra. The base of the penis was mobilized a few centimeters to enable penile fixation sutures at the base. The urethral and dorsal neurovascular bundle was identified and protected. The area of penile skin deficit was measured. Elliptical incisions were made in the bilateral groin creases to FTSG, which was then defatted. The FTSG was spirally inserted into the penile shaft, and Tisseel fibrin sealant (4 cc) was used. An Adaptic dressing and sponge gauze were applied and secured to the abdominal skin by prolene sutures. Groin closure was completed using staples.

No early postoperative complications were reported, and the patient's vital signs and laboratory investigations were normal. The penile dressing was kept dry, and the penile glans were healthy with preserved sensation. The patient's hips were kept flexed to reduce tension in his groin wounds. Meropenem and clindamycin were continued.

**Fig. 3:**
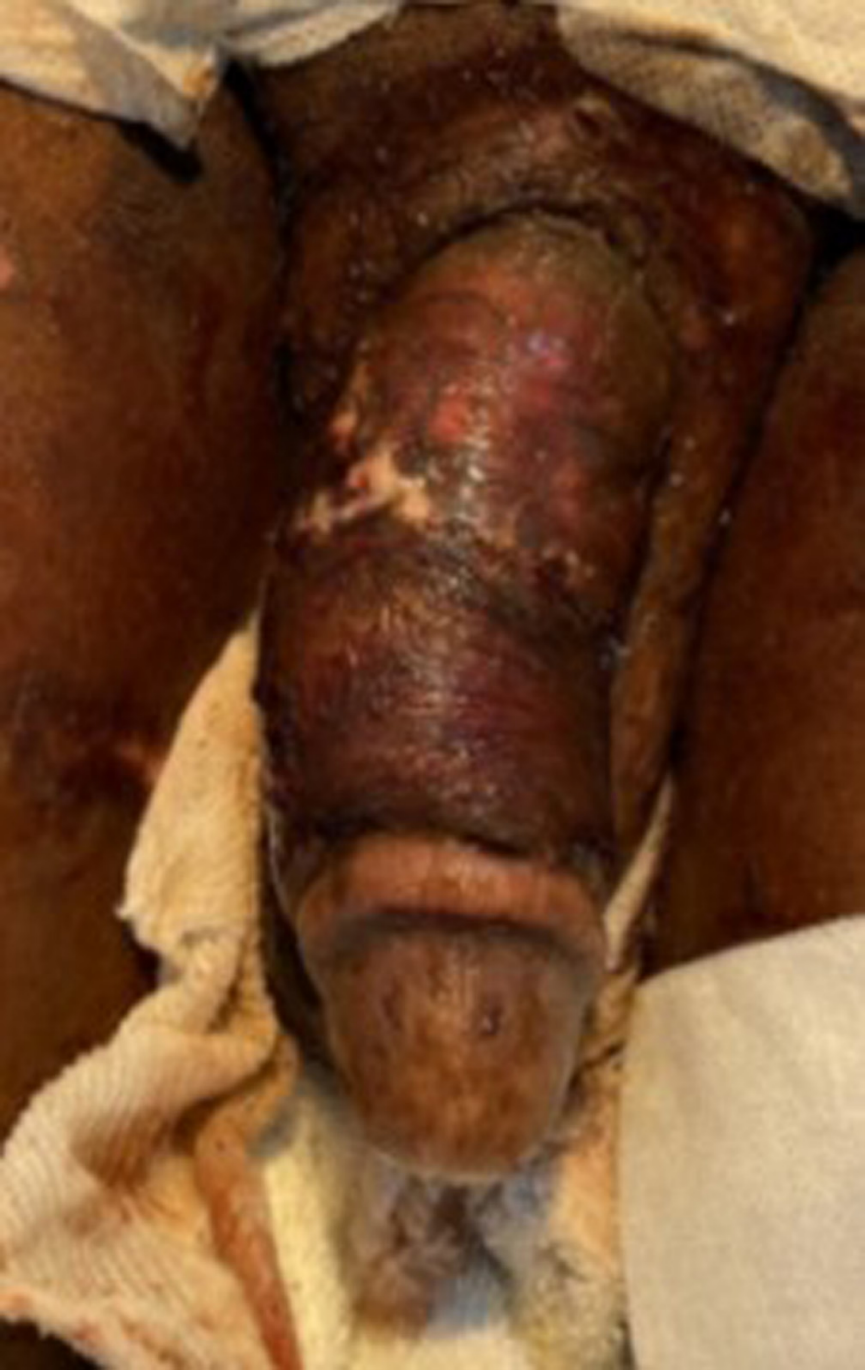
One week after the graft operation, the penile wound appeared clean and was healing.

After a 20‐day hospital admission, the patient was discharged back to the prison without antibiotics. The patient was clinically and vitally stable with clean wounds, which were healing (Fig. 3). His laboratory investigations were normal, and a leg bag was attached to the SPC. The wound management plan was given to the prison medical team.

## Discussion

Penile strangulation can be caused by metallic or non‐metallic objects, such as metal rings, plastic bottlenecks, and rubber bands. The strangulated object can block venous drainage alone or concurrent arterial supply and venous drainage. Blockage of both systems may cause ischemic penile necrosis. Obstruction of the venous drainage alone may cause lymphedema and subsequent penile compartment syndrome, which may lead to secondary necrosis.^3^

The flexible properties of non‐metallic objects allow for easier positioning and removal. Nevertheless, this may cause more tissue injury due to a greater compressive effect on the penis during the edema phase.^4^

Penile strangulation may cause different complications depending on the type of object used, along with the degree and duration of the constriction. Bhat and colleagues established a classification system for penile strangulation injuries, which was simplified and modified by Silberstein *et al*. (Table 1).^1^, ^5^

**Table 1:** Grading and management of penile strangulation injuries^1^, ^5^

Bhat grading system	Silberstein revised grading system	Management
Grade 1: Edema of the distal penis. No evidence of skin ulceration or urethral injury	Low‐grade penile injury	Are likely to require no further intervention once the constricting object has been extricated.
Grade 2: Injury to skin and constriction of corpus spongiosum but no evidence of urethral injury. Distal penile edema with decreased penile sensation		
Grade 3: Injury to skin and urethra but no urethral fistula. Loss of distal penile sensation		
Grade 4: Complete division of corpus spongiosum leading to urethral fistula and constriction of corpus cavernosum with loss of distal penile sensation	High‐grade penile injury	Are likely to require surgical intervention.
Grade 5: Gangrene, necrosis, or complete amputation of the distal penis		

Our case was considered a Grade V Bhat grade or high‐grade injury in the Silberstein grades because of the penile necrosis and surgical intervention.

Management of the strangulated penis depends on the patient's presentation. If the strangulating object is metallic, the patient may present with the object in place. However, if the object is non‐metallic, the patient will be more likely to present with subsequent complications of strangulation. The patient may present with different degrees of penile injury (Table 1).

## Conclusions

Timely management of penile strangulation is crucial to prevent vascular and mechanical complications. This emphasizes the importance of promptly addressing penile strangulation and the possible complications it may entail. It is advisable for penile strangulation patients to undergo a psychological assessment to assess any potential underlying psychiatric disorders that might have played a role in the incident.

## Author contributions

Abdulla Uthman: Methodology; project administration; supervision; writing – original draft; writing – review and editing. Shahad Al‐Mashhadani: Writing – original draft; writing – review and editing. Naomi Blower: Writing – original draft. Muhammed Iqbal: Supervision; writing – review and editing.

## Conflict of interest

None.

## Approval of the research protocol by an Institutional Reviewer Board

Not applicable.

## Informed consent

The patient gave written informed consent to use his pictures for research publication.

## Registry and the Registration No. of the study/trial

Not applicable.
